# Semiconducting Electrides Derived from Sodalite: A First-Principles Study

**DOI:** 10.1021/acsomega.4c09513

**Published:** 2025-01-06

**Authors:** Chang Liu, Musiha Mahfuza Mukta, Byungkyun Kang, Qiang Zhu

**Affiliations:** † Department of Physics and Astronomy, University of Nevada, Las Vegas, Nevada 89154, United States; ‡ Department of Mechanical Engineering and Engineering Science, University of North Carolina at Charlotte, Charlotte, North Carolina 28223, United States; § College of Arts and Sciences, University of Delaware, Newark, Delaware 19716, United States; ∥ International Center for Computational Methods & Software, College of Physics, Jilin University, Changchun, Jilin 130012, China

## Abstract

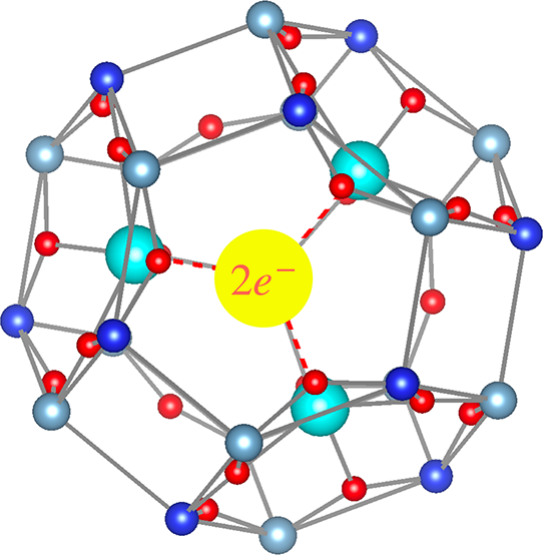

Electrides are ionic crystals, with electrons acting as anions occupying well-defined lattice sites. These exotic materials have attracted considerable attention in recent years for potential applications in catalysis, rechargeable batteries, and display technology. Among this class of materials, electride semiconductors can further expand the horizon of potential applications due to the presence of a band gap. However, there are only limited reports on semiconducting electrides, hindering the understanding of their physical and chemical properties. In recent work, we initiated an approach to derive potential electrides via selective removal of symmetric Wyckoff sites of anions from existing complex minerals. Herein, we present a follow-up effort to design semiconducting electrides from parental complex sodalites. Among four candidate compounds, we found that a cubic Ca_4_Al_6_O_12_ structure with the *I*-43*m* space group symmetry exhibits perfect electron localization at the sodalite cages, with a narrow electronic band gap of 1.8 eV, making it suitable for use in photocatalysis. Analysis of the electronic structures reveals that a lower electronegativity of the surrounding cations drives greater electron localization and promotes the formation of an electride band near the Fermi level. Our work proposes an alternative approach for designing new semiconducting electrides under ambient conditions and offers guidelines for further experimental exploration.

## Introduction

Electrides are exotic ionic materials in which electrons occupy well-defined lattice sites and serve the role of anions. These materials were originally discovered by Dye and coworkers in organic salts in the 1980s; however, their high sensitivity to heat and oxidation limits their practical application.^1,2^ Subsequent efforts have been directed toward achieving stable electrides, aiming to explore chemical and physical properties arising from nearly free electrons and their corresponding geometric topologies. In 2003, the first thermally stable electride, Ca_6_Al_7_O_16_ (C12A7:2e^–^) was synthesized from the inorganic mineral mayenite (12CaO·7Al_2_O_3_).^3^ The resulting electride, with excess electrons confined in zero-dimensional (0D) cages, exhibits excellent thermal stability and low reactivity with air. This discovery marks a milestone in the research on electrides and spurred numerous new efforts to design other novel inorganic electrides. To date, electrides have been identified in various inorganic compounds exhibiting different configurations of confined electrons,^4,5^ including 0D cavities,^3,6,7^ 1D chains,^8−12^ 2D planes,^13−17^ and 3D configurations.^18^ These diverse electronic configurations produce unique properties, including high electron concentration,^13,19^ antiferromagnetism,^11^ thermionic electron emission at low temperature,^13,20^ high density of active sites,^21^ and low work function.^22^ These exceptional properties make electrides promising candidates for applications in catalysis,^23,24^ energy storage,^25,26^ and electronics.^27,28^

While most currently reported electrides are semimetals, an electride with a semiconducting band structure has the potential to further expand the horizon of potential applications of such electrides as infrared photodetectors.^29^ A previous theoretical study proposed a transition in Ca_2_N from a 2D conducting electride to a 0D semiconducting electride state under high pressure.^30^ This discovery was supported by electrical resistance measurements conducted in a follow-up experimental study.^31^ To further investigate the physical and chemical properties and explore possible applications of semiconducting electrides, stable structures at ambient conditions still need to be developed.^32^ In 2022, a novel inorganic electride, Sc_2_C, was discovered as the first 2D electride exhibiting semiconducting behavior.^29^ Its small band gap and good conductivity enable applications as a battery electrode or in IR photodetectors. A recent theoretical study identified Mg_2_N in *R*3*m* symmetry as a 0D electride with a semiconducting band structure.^33^ However, research on this novel class of electrides is still in its infancy. Consequently, discovering new semiconducting electrides with different confined electron configurations stabilized at ambient conditions is essential to understand related properties and new electride design toward potential applications.

**Figure 1:**
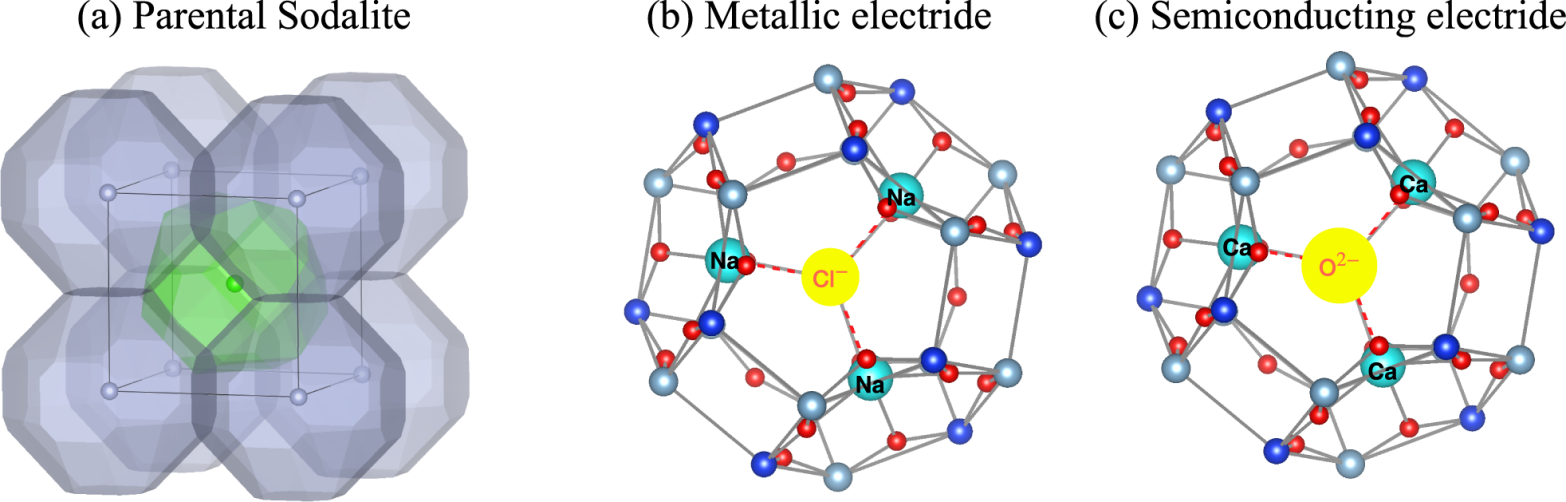
Schematic illustration of electride properties.

Recently, we introduced an alternative method to achieve electride states by removing high-symmetry Wyckoff sites of anions from existing sodalite compounds.^34^ The resulting sodalite electrides are expected to exhibit greater thermal stability due to their intricate structural framework, thereby holding promise for practical applications. In our previous work, we primarily focused on halides, where only one electron can be accommodated per cage in the resulting sodalite framework (Figure 1). Consequently, the localized cage electrons can lead to either a half-metallic state or a Mott insulator from the perspective of electron counts.^34^ According to the electron counting rule, it is likely that two localized electrons in a cage of the crystal structure may form a distinct electronic band with full occupation, leading to a semiconducting or an insulating band structure. Although this simple model may be complicated by the interplay between the cage electrons and their surrounding cations, we hypothesize that this is a viable approach to designing new electride materials with a nonzero band gap. Therefore, we have decided to further explore this avenue by investigating sodalite compounds that contain high-symmetry Wyckoff sites of anions with a formal charge of -2.

In this work, we applied a screening strategy similar to our previous study^34^ on existing multicomponent sodalites and identified four candidate parental structures characterized by the presence of chalcogen anions in high-symmetry Wyckoff sites. These candidates include Ca_4_Al_6_O_13_, Zn_4_B_6_O_13_, Cd_4_Al_6_SO_12_, and Be_3_Cd_4_Si_3_SeO_12_. We initially examined the lattice dynamical properties of these structures after the removal of the anionic site at the center of the unit cell. Theoretical results indicate that the generated compounds are dynamically stable under ambient conditions. Subsequently, we conducted electronic structure calculations on these derivative compounds to verify the presence of the electride states. Interestingly, we found that these sodalite compounds, with various metal cations adjacent to the electride site, provide an ideal platform for exploring systematic trends. Our further analysis of the evolution of electronic properties with the adjacent cations revealed that a lower electronegativity of the adjacent cation can stabilize the electride states and generate a distinct electride band around the Fermi level.

## Computational Methods

Following a query of the Materials Project^35^ using our in-house symmetry analysis toolbox, PyXtal,^36^ we selected four complex sodalite structures in cubic symmetry: Ca_4_Al_6_O_13_, Zn_4_B_6_O_13_, Cd_4_Al_6_SO_12_ in *I*-43*m* space group, and Cd_4_Be_3_Si_3_SeO_12_ in *P*43*n* space group as parent structures to create the electride phases. For each structure, the electride configuration was generated by removing the anions from the center of the unit cell. The structures were then fully relaxed to obtain optimal cell parameters, and their structural stability was checked using phonon calculations.

All calculations were performed using the projector augmented wave (PAW) method,^37^ implemented in the VASP code^38^ within the framework of density functional theory (DFT). The generalized gradient approximation (GGA) with the Perdew, Burke, and Ernzerhof (PBE) functional^39^ was adopted. Optionally, we also considered the inclusion of the nonlocal van der Waals functional (PBE+rVV10L) to investigate the impact of van der Waals dispersion.^40^ For geometric relaxation, we used a unit cell containing two formula units, and a 3 × 3 × 3 Γ-centered *k*-point grid was adopted. To simulate electronic properties, a primitive cell with a dense mesh of an 8 × 8 × 8 *k*-point grid was utilized. The cutoff energy for all calculations was set to 520 eV, achieving convergence for energy around 1 meV per atom and for forces within 0.01 eV/Å. In addition to the PBE functional, the band gap values were recomputed by using the HSE06 hybrid functional.^41^ For phonon calculations, the optimized structures for electrides were used to construct either a 2 × 2 × 2 supercell (containing 176 atoms) or a 3 × 3 × 3 supercell (containing 594 atoms), employing a single Γ point for Brillouin zone sampling. Phonon band dispersion relations were then calculated using force constants obtained via the finite displacement method as implemented in the Phonopy code.^42^ To further confirm the thermal stability, we also ran at least 10 ps molecular dynamics simulations using the on-the-fly machine-learning algorithm^43^ for some selected structures under the NPT ensemble.

## Results and Discussions

### Crystal Structures and DFT Benchmarks

**Figure 2:**
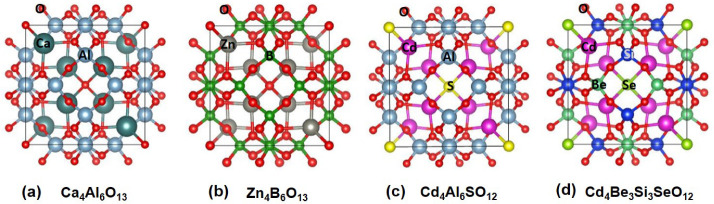
Crystal structures of (a) Ca_4_Al_6_O_13_, (b) Zn_4_B_6_O_13_, (c) Cd_4_Al_6_SO_12_, and (d) Cd_4_Be_3_Si_3_SeO_12_. The high symmetry cage anion sites are located at the body center position in each structure.

In this work, all four candidate parental oxides have already been observed in experiments.^44−47^ As shown in Figure 2, each unit cell contains two formula units. According to X-ray diffraction analysis,^44^ Ca^2+^ is bonded to four O^2–^ atoms to form distorted CaO_4_ trigonal pyramids that share corners with six equivalent AlO_4_ tetrahedra and corners with three equivalent CaO_4_ trigonal pyramids. Other materials, including Zn_4_B_4_O_13_, Cd_4_Al_4_SO_12_, and Cd_4_Be_3_Si_3_SeO_12_ show similar packing behavior.

Table 1 summarizes the calculated cell parameters at both PBE and PBE+rVV10L levels and the band gap values at both PBE and HSE06 levels. In general, we expect that more advanced functional choices, such as the HSE06 hybrid functional, vdW-inclusive models, or more recently developed self-interaction-corrected methods,^48,49^ should potentially yield more accurate predictions of band structure and crystal geometry. According to Table 1, we indeed found that PBE+rVV10L improves the overall lattice parameter agreement and HSE06 systematically increases the band gap value, as compared to the PBE results. However, the computed electronic structure and chemical bonding picture remain the same. Since the main focus of our work is to provide an intuitive guide for the design of semiconducting electrides, we choose to adopt the standard PBE functional for the remainder of our discussions. Further refinement of numerical modeling will be pursued in future studies if experimental confirmation of the predicted compounds is achieved.

**Table 1:** Crystallographic Data and Band Gaps of Four Parent Sodalites and Their Derivative Electride Candidate Structures

System	Symmetry	*a* (Å)			Band gap (eV)	
		PBE	VDW	Expt.	PBE	HSE06
Sodalites						
Ca_4_Al_6_O_13_	*I*-43*m*	8.87	8.77	8.86^44^	3.9	5.5
Zn_4_B_6_O_13_	*I*-43*m*	7.55	7.46	7.47^45^	3.4	5.2
Cd_4_Al_6_SO_12_	*I*-43*m*	8.95	8.82	8.82^46^	2.7	4.2
Cd_4_Be_3_Si_3_SeO_12_	*P*43*n*	8.62	8.50	8.49^47^	2.9	
Electride candidates						
Ca_4_Al_6_O_12_	*I*-43*m*	9.06	8.92		1.2	1.8
Zn_4_B_6_O_12_	*I*-43*m*	7.41	7.29		3.6	5.0
Cd_4_Al_6_O_12_	*I*-43*m*	8.64	8.44		2.8	3.8
Cd_4_Be_3_Si_3_O_12_	*P*43*n*	8.22	8.06		3.0	4.2

For each structure, we removed the cage anions at the body center of the unit cells to generate candidate electride structures. Specifically, we removed the O^2–^ for Ca_4_Al_6_O_13_ and Zn_4_B_6_O_13_, S^2–^ for Cd_4_Al_6_SO_12_, and Se^2–^ for Cd_4_Be_3_SeO_12_. Correspondingly, the resulting candidate electride structures consist of cages surrounded by four Ca^2+^ in Ca_4_Al_6_O_12_, four Zn^2+^ in Zn_4_B_6_O_12_, and four Cd^2+^ in both Cd_4_Al_6_O_12_ and Cd_4_Be_3_Si_3_O_12_. Comparing the cell parameters between the parental and anion-removal phases, we found that most of the systems undergo a lattice shrinkage after the removal of anions (see Table 1). However, Ca_4_Al_6_O_12_ is an exception. After the removal of O^2–^, the volume unexpectedly increases, suggesting that new types of interactions may form due to the localized cage electrons.

### Spatial Electron Localization

To characterize the electride state, we calculated the electron localization function (ELF), which measures the degree of an electron’s spatial localization compared to a reference electron with the same spin. The ELF value, bounded between 0 and 1, reflects the likelihood of finding an electron in the neighborhood of a reference electron located at a given point, with ELF = 1 corresponding to perfect localization and ELF = 0.5 corresponding to a free electron gas.^50^

**Figure 3:**
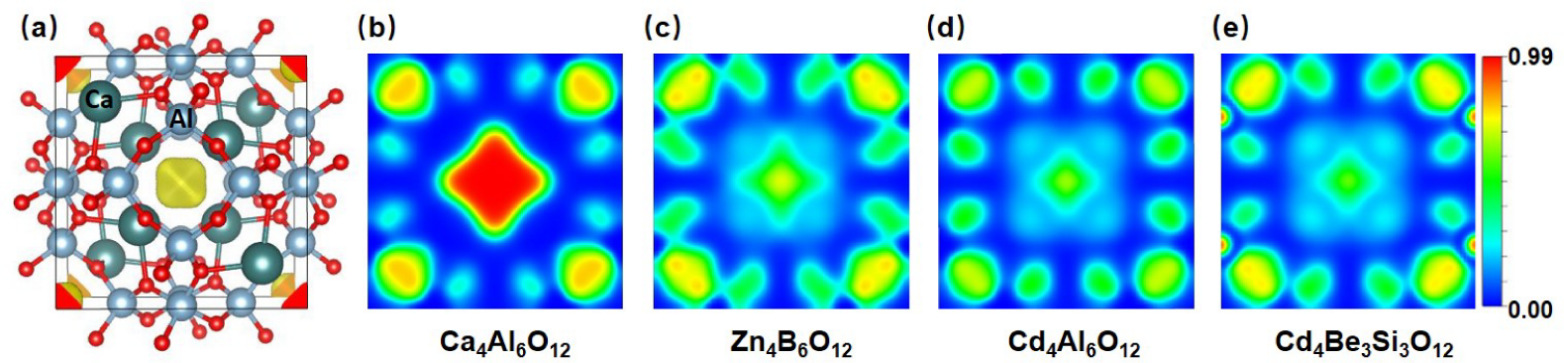
Calculated electron localization function (ELF) after removal of the phosphorus atom from the solution of O^2–^. (a) 3D ELF isosurface ELF of Ca_4_Al_6_O_12_. (b-e) 2D ELF of Ca_4_Al_6_O_12_, Zn_4_B_6_O_12_, Cd_4_Al_6_O_12_, and Cd_4_Be_3_Si_3_O_12_ in the (200) plane.

As shown in Figure 3, we observed strong ELF values at the body center cages after the removal of the cage anions from the parent structures. These results suggest that the electrons remain localized at the sodalite cages. Among them, Ca_4_Al_6_O_12_ possesses an ELF value of 0.99 at the body center, suggesting the strongest electron localization. On the other hand, the ELF values at the cage centers for Zn_4_B_6_O_12_, Cd_4_Al_6_O_12_, and Cd_4_Be_3_Si_3_O_12_ are 0.72, 0.65, and 0.59, respectively. The trend of the ELF values has a strong correlation with the electronegativity of the surrounding metal cations. Ca has an electronegativity value of 1.00, whereas Zn has a value of 1.65 and Cd of 1.69.^51^ Due to its lower electronegativity compared to Zn^2+^ and Cd^2+^ cations, Ca^2+^ is expected to have a weaker attraction to the outer core electrons, consequently leading to greater electron localization at the body center cages in Ca_4_Al_6_O_12_.

### Electronic Band Structures

**Figure 4:**
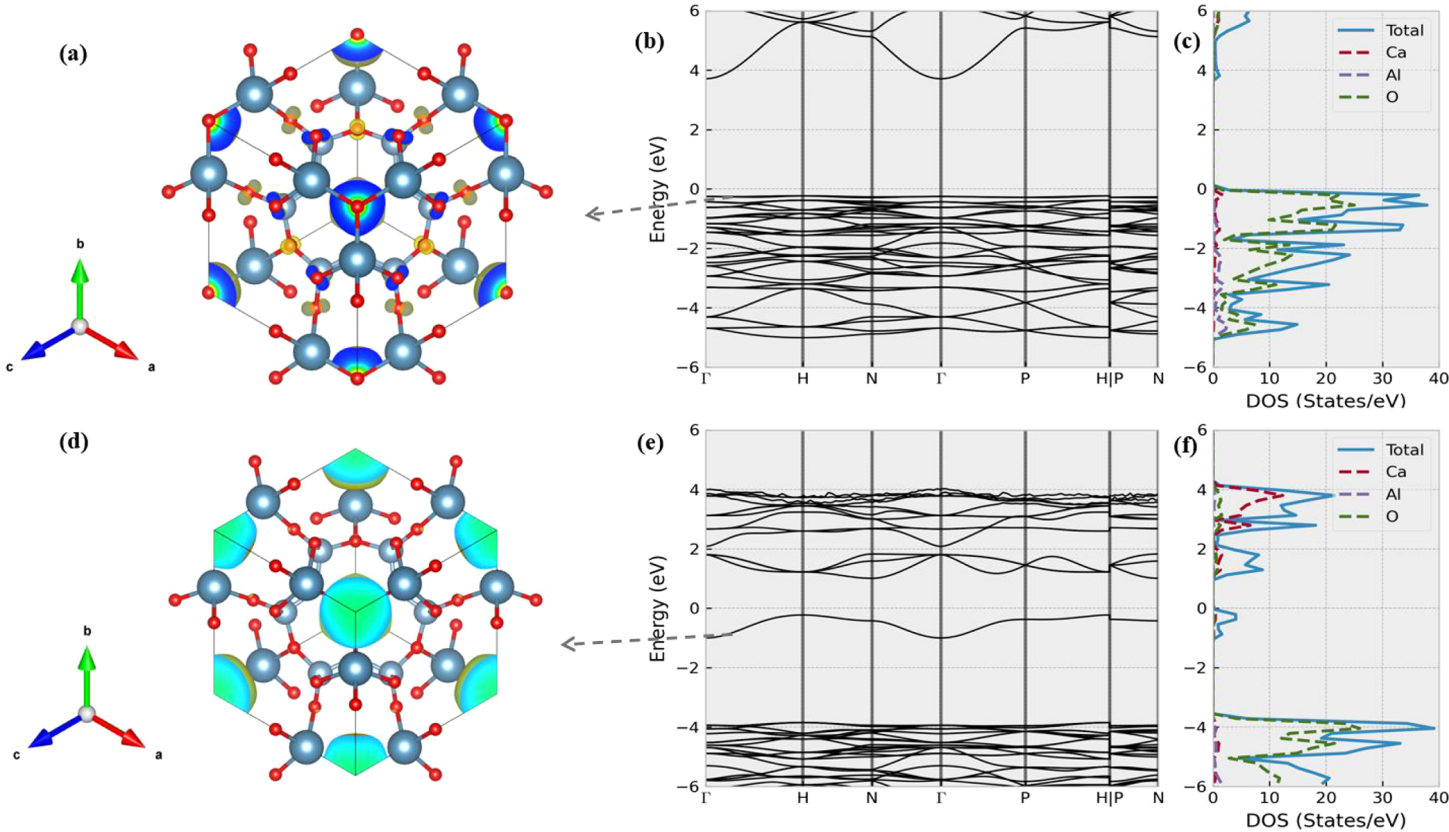
Electronic structures of Ca_4_Al_6_O_13_ and Ca_4_Al_6_O_12_. (a) displays the isosurface of the decomposed charge density of Ca_4_Al_6_O_13_’s highest valence band, whereas (b) and (c) plot its band dispersion and density of states (DOS) at an extended energy range around the Fermi level. As a comparison, (d–f) show the isosurface of the decomposed charge density of Ca_4_Al_6_O_12_’s highest conduction band and its corresponding band structure and DOS plots. The isosurface value is set as 0.003 e/bohr^3^ in parts (a) and (d).

In addition to the electron’s spatial localization, we are also interested in understanding their electronic band structures and the energy levels of those localized cage electrons. As shown in Figure 4b, the parent structure Ca_4_Al_6_O_13_ is a semiconductor with a band gap of 3.9 eV, and the highest valence band (HVB) near the Fermi level is very flat. The associated DOS plot (Figure 4c) suggests that the HVB is mainly contributed by the O orbitals, while the conduction bands comprise the hybridization of oxygen and metal atoms. More precisely, we can clearly see that the decomposed charge density of the HVB is mainly featured by the electrons around the O^2–^ anions at the cage center, as shown in Figure 4a. Overall, the HVB in Ca_4_Al_6_O_13_ has an energy close to that of other valence bands.

After the removal of cage O^2–^ anions, the resulting structure maintains a semiconducting band structure with a much narrower band gap of 1.2 eV. As shown in both Figure 3d,e, the Ca_4_Al_6_O_12_ has a HVB clearly separated from other valence bands in the system. Remarkably, the computed partial charge configuration in Figure 4d, unlike what is shown in Figure 4a, suggests that this band solely corresponds to electrons localized at cage centers.

In our previous work on the screening of sodalite halide electride,^34^ we found that only one electron is confined in each cage after the removal of halide atoms, thus leading to a partially occupied energy band for the interstitial electrons. According to our earlier proposal based on the electron counting rule, the physical picture should change substantially if the oxygen or sulfur atoms were removed. When O is present at the cage center, each O has the capability to attract excess electrons from four neighboring Ca atoms, leading to a charge transfer from Ca to O and forming the ionic bonding between O^2–^ and Ca^2+^. This ionic bonding can also help to lower the valence band energy. When the caged O atoms are removed, the excess electrons from the Ca atoms cannot be redistributed to any surrounding cations. As a result, the localized cage electrons behave like nucleus-free anions to stabilize the crystal structure. If there is only one electron in each cage, the system should form a partially occupied band as discussed in our previous work.^34^ Given that two electrons are present in each cage of Ca_4_Al_6_O_12_, these electrons form a fully occupied electronic band, and the whole structure remains semiconducting. However, this band is expected to possess a higher energy compared to the counterpart HVB in Ca_4_Al_6_O_13_. Notably, the repulsion between localized cage electrons and surrounding Ca^2+^ in Ca_4_Al_6_O_12_ also tends to expand the [Ca]_4_ tetrahedra and thus leads to a larger volume as compared to the parental phase. Therefore, the band gap in Ca_4_Al_6_O_12_ becomes smaller, and we can safely conclude that Ca_4_Al_6_O_12_ is a typical electride feature with a semiconducting band gap. To our knowledge, this is the first report of a non-layered electride phase that can be stabilized at ambient pressure conditions.

**Figure 5:**
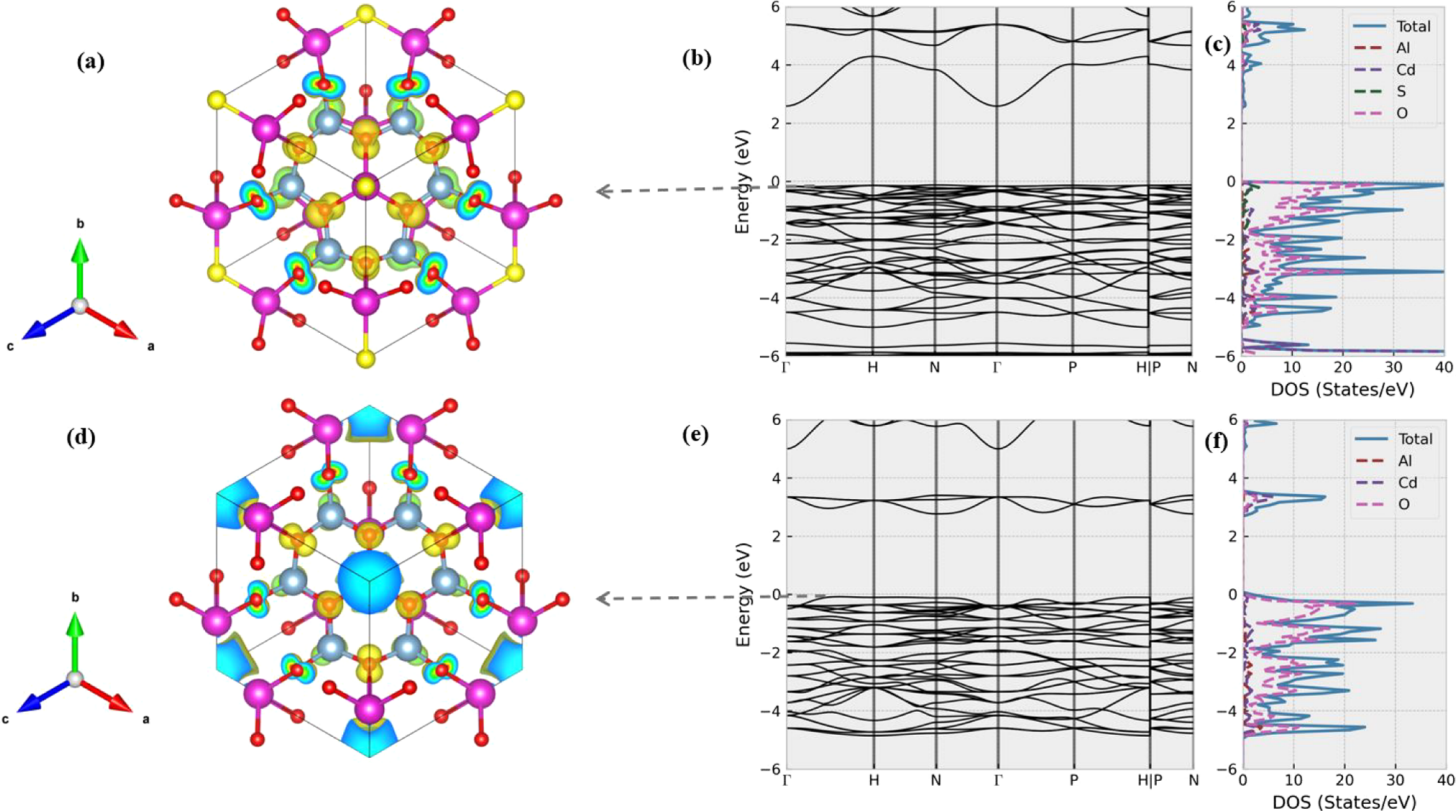
Electronic structures of Cd_4_Al_6_SO_12_ and Cd_4_Al_6_O_12_. (a) displays the decomposed charge density of Cd_4_Al_6_SO_12_’s highest valence band, whereas (b,c) plot its band dispersion and DOS at the extended range. As a comparison, parts (d–f) show the isosurface of the decomposed charge density of Cd_4_Al_6_O_12_’s highest valence band, as well as its corresponding band structure and DOS plots. The isosurface value is set as 0.003 e/bohr^3^ in (a) and (d).

While the case of Ca_4_Al_6_O_12_ demonstrates the possibility of achieving an energy band fully occupied by interstitial electrons near the Fermi level, the interplay with the local chemical environment may complicate this scenario. As shown in Figure 5, the parent system Cd_4_Al_6_SO_12_ is a semiconductor with a band gap of 2.7 eV. The top valence bands near the Fermi level of Cd_4_Al_6_SO_12_ mainly come from the O, S, and Cd atoms. Upon removing the S atoms, we can find several high-energy conduction bands drop off, similar to those in Ca_4_Al_6_O_12_. However, the band gap of Cd_4_Al_6_O_12_ does not vary significantly from that of the parent structure. This is also evidenced by the visualization of the partial charge density of the valence band closest to the Fermi level (see Figure 5d). While there exist localized electrons around the cage center, we also find a significant portion of electrons around the neighboring O atoms (that are similar to the parental compound). Therefore, we can better interpret this band as reflecting an interaction between Cd_4_ cluster cations and neighboring O anions. Compared to the cage electrons in Ca_4_Al_6_O_12_, the cage electrons in Cd_4_Al_6_O_12_ are tightly bound by the Cd nucleus. Therefore, the charge redistribution after the removal of S atoms in Cd_4_Al_6_SO_13_ fails to generate an isolated energy band fully occupied by interstitial electrons near the Fermi level. We also observed a similar trend in Cd_4_Be_3_Si_3_SeO_12_ (see Figure S2). In this system, the corresponding partial charge density plot in Cd_4_Be_3_Si_3_O_12_ reveals that O contributes more portions compared to the cage electrons, suggesting that the substitution of Be and Si can systematically shift the charge redistribution.

**Figure 6:**
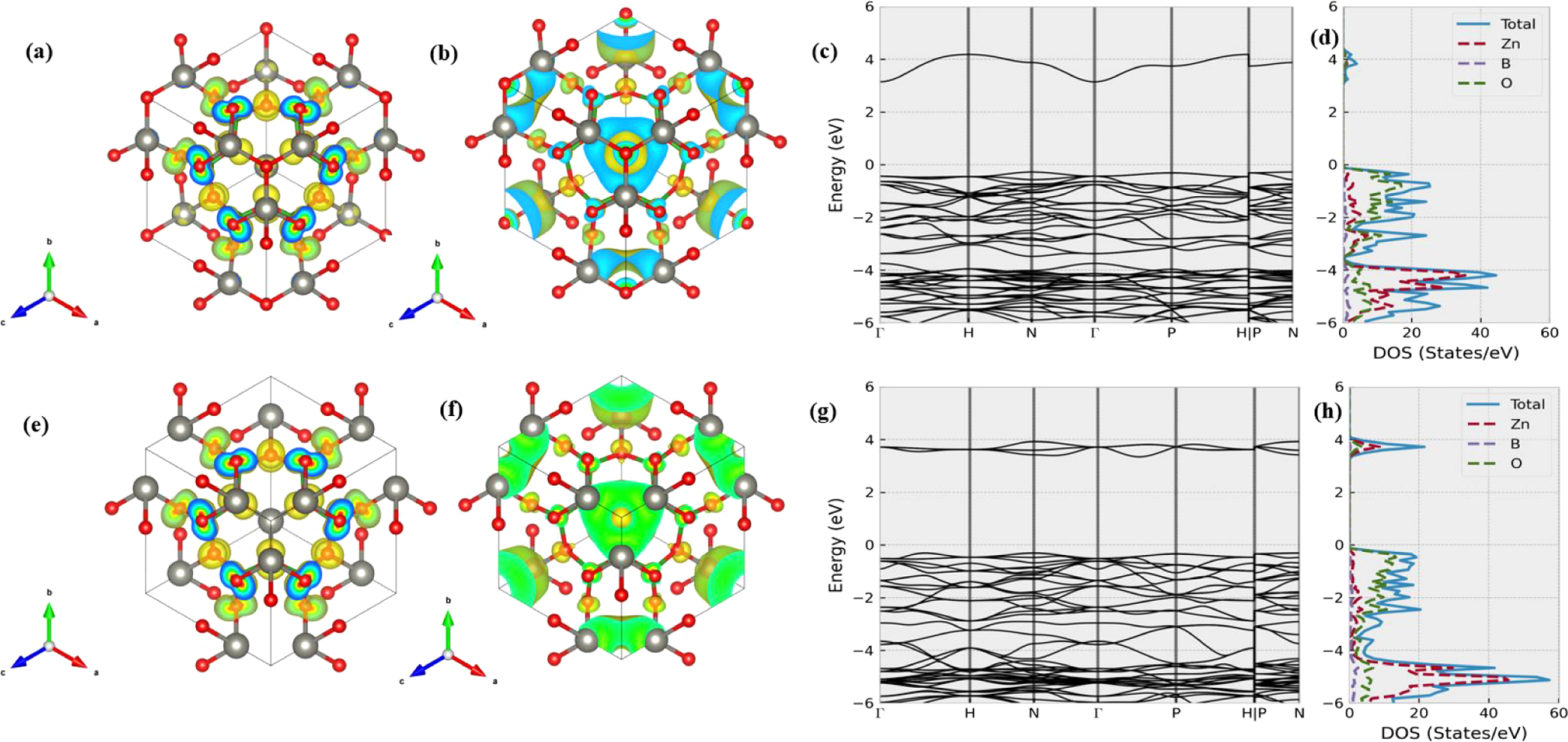
Electronic structures of Zn_4_B_6_O_13_ and Zn_4_B_6_O_12_. (a) and (b) display the decomposed charge densities of Zn_4_B_6_O_13_’s highest valence band and lowest conduction band, whereas (c) and (d) plot its band dispersion and DOS at the extended range. As a comparison, (e–h) show the isosurface of the decomposed charge density of Zn_4_B_6_O_12_’s highest valence band, lowest conduction band, the full band structure, and DOS plots. The isosurface value is set as 0.003 e/bohr^3^ in (a), (b), (e), and (f).

As shown in Figure 6, Zn_4_B_6_O_13_ is a semiconductor with a band gap of 3.9 eV. Its valence bands near the Fermi level are contributed by Zn and noncage O atoms. Following the removal of caged O atoms, we observe a lowering of several high-energy conduction bands, similar to what is found in other systems. According to Figure 6f, these bands correspond to the antibonding states with localized electrons around the cage. However, we do not find a significant electron density around the cage in the top valence bands. Instead, the HVB of Zn_4_B_6_O_13_ in Zn_4_B_6_O_12_ mainly consists of electrons around noncage O and Zn atoms (see Figure 6e). This suggests that the majority of excess electrons tend to return to the Zn atoms rather than remain localized around the cage. Consequently, the band gap of Zn_4_B_6_O_12_ does not vary significantly from that of the parent structure.

**Figure 7:**
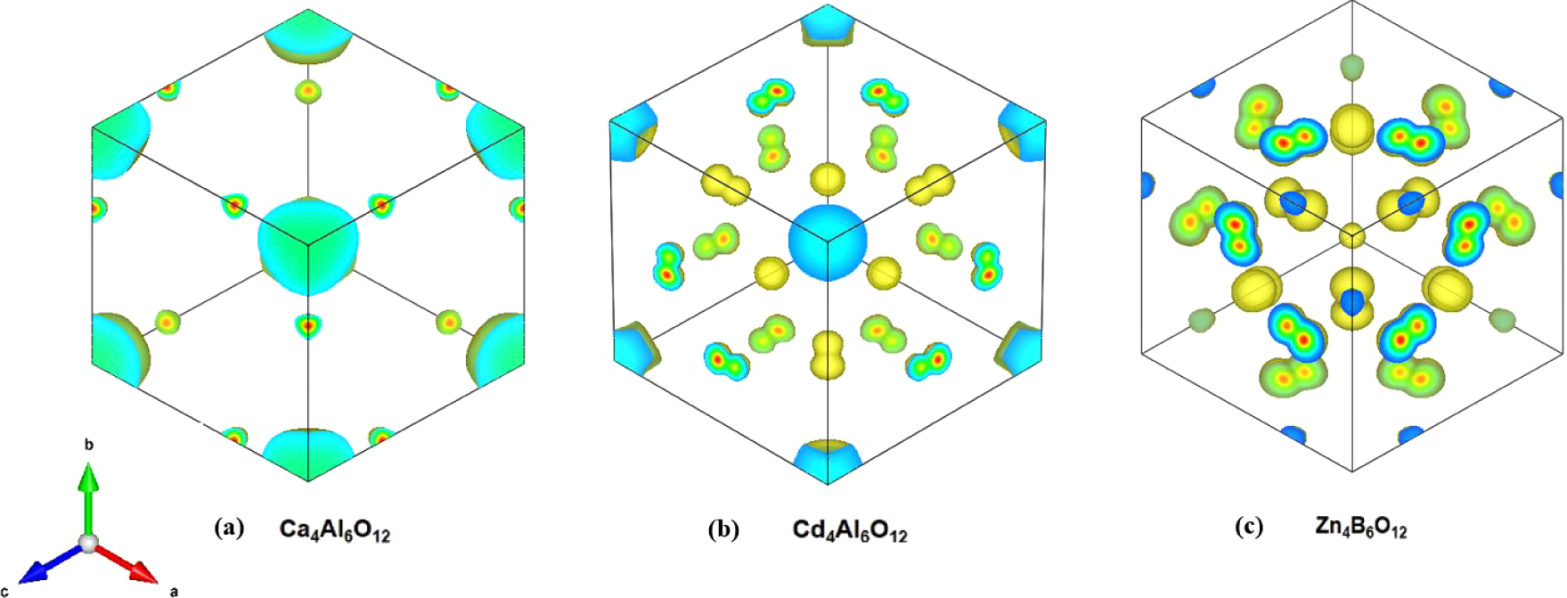
Comparison of charge redistributions after the removal of cage atoms in the (a) Ca_4_Al_6_O_12_, (b) Cd_4_Al_6_O_12_, and (c) Zn_4_B_6_O_12_. In order to track the charge density near the atomic nucleus, we intentionally omitted the atomic spheres for clarity. The isosurface value is set as 0.004 e/bohr^3^ to remove some very small charge densities.

Finally, we present the partial charge distribution in the top valence bands of Ca_4_Al_6_O_12_, Cd_4_Al_6_O_12_, and Zn_4_B_6_O_12_ in Figure 7, where atomic spheres in the unit cell have been removed for clarity. These plots illustrate how the two excess electrons are redistributed following the removal of the cage atoms. In Ca_4_Al_6_O_12_, the high valence band (HVB) charge density indicates that the excess electrons remain localized within the cage. However, this arrangement is energetically unfavorable, leading to an upward shift of the HVB in its band structure and a consequent reduction in the band gap. Upon replacing Ca with Cd, the electronic behavior in Cd_4_Al_6_O_12_ changes significantly. Due to Cd’s higher electronegativity, a portion of the cage electrons is redistributed toward the Cd nucleus, as shown in Figure 7b. In Zn_4_B_6_O_12_, the charge redistribution is even more pronounced (see Figure 7c). Here, the majority of excess electrons accumulate around the Zn nucleus due to the attractive forces from both neighboring Zn and B nuclei. In both Zn_4_B_6_O_12_ and Cd_4_Al_6_O_12_, this charge redistribution contributes to maintaining a low-energy state, resulting in no significant band gap reduction in these systems.

From the above analysis, we find that after removing the cage center atoms in the sodalite structures, all systems may accommodate electrons at the cage center. However, these electrons may not be sufficient to generate a fully occupied energy band near the Fermi level. The electronegativity of the cations adjacent to the cage plays a crucial role in this phenomenon. Among the four systems studied, only the cage electrons surrounded by the electron-rich Ca^2+^ cations form a fully occupied valence band near the Fermi level. These electrons create an anionic electronic sphere that interacts weakly with the neighboring Ca^2+^ cations. In contrast, the cage electrons in the other systems may be redistributed to the neighboring Cd^2+^ or Zn^2+^ cations, preventing them from being considered as nucleus-free anions. This observation aligns with our previous screening work,^5^ which indicated that only group I, II, and early transition metals can form electrides.

### Stability and Experimental Synthsizability

We computed the phonon spectrum for each structure with a 2 × 2 × 2 supercell setting and found that none exhibit imaginary frequencies (see Figure S1). These results indicate that all structures remain dynamically stable after the removal of the cage anions.

**Figure 8:**
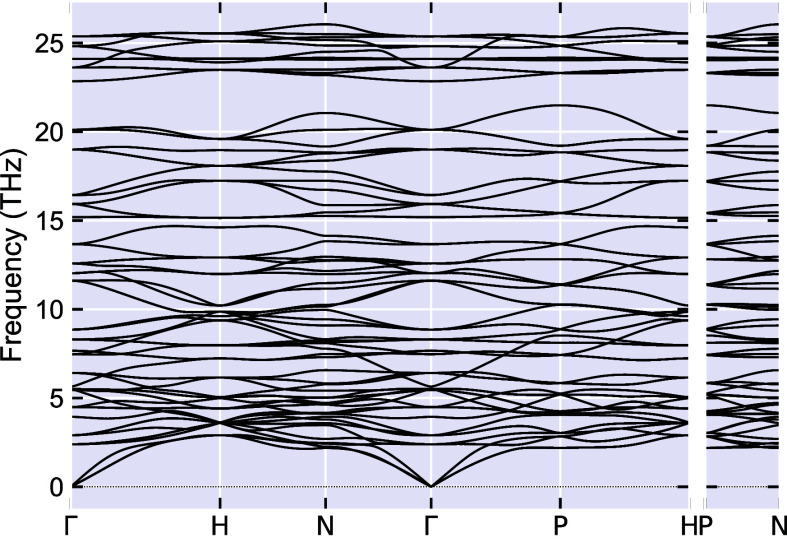
Calculated phonon band structure for Ca_4_Al_6_O_12_ with a 3 × 3 × 3 supercell.

For the most important results for the semiconducting Ca_4_Al_6_O_12_ electride, we also considered a 3 × 3 × 3 supercell in our phonon calculation. As shown in Figure 8, the results are very similar to those in Figure S1, thus confirming our phonon calculation based on the 2 × 2 × 2 supercells.

To check the thermal stability, we also performed a 10 ps molecular dynamics simulation for a 2 × 2 × 2 Ca_4_Al_6_O_12_ supercell under the NPT ensemble at 500 K and standard pressure conditions (see Figure S3). The results reveal that the system quickly reaches equilibrium and then just fluctuates around it without significant structural and energy changes, suggesting that the structure is indeed stable upon thermal fluctuations up to 500 K and perhaps even at higher temperatures.

Given that the parent compound has already been found in the experiment, it is expected that a minor change should not destroy the structural stability. Therefore, Ca_4_Al_6_O_12_, together with other candidates, should have good stability and persist as long as they are synthesized under experimental conditions. However, the experimental realization still depends on the synthesis pathway of the parental compounds^44^ that needs to be evaluated from different perspectives.

## Conclusions

In this work, we conducted a survey to design new semiconducting electrides by selectively removing anions from existing sodalite structures. Our simulations reveal notable electron localization near the cage center after the removal of anions occupying the high-symmetry Wyckoff sites in the sodalite structures. However, the localized cage electrons may or may not form a distinct electride state in the electronic band structure, primarily depending on the electronegativity of the surrounding cations near the sodalite cages. Different from our earlier work on the metallic electrides derived from halide sodalites,^34^ we found that the sodalite electrides derived from oxides and sulfides possess a semiconducting band structure due to the localization of two electrons in the cage. Among the candidate compounds, Ca_4_Al_6_O_12_ serves as an ideal example of electron localization capable of forming a fully occupied energy band near the Fermi level. This leads to a semiconducting electride with a significantly reduced HSE06 band gap of 1.8 eV, compared to the parental sodalite structure’s band gap of 5.5 eV. Additionally, this phase may exhibit improved thermal stability due to its complex structural framework. We hope that our findings will guide further experimental designs for new semiconducting electrides.

## Supporting Information Available

Calculated phonon spectra for all compounds; the electronic structures of the parent structure Cd_4_Be_3_Si_3_SeO_12_ and Cd_4_Be_3_Si_3_SiO_12_; and the MD simulation results (PDF)
