# Supplementary material for: Semiconducting Electrides Derived from Sodalite: A First-Principles Study
Source: ACS Omega. 2025 Jan 6;10(1):1635–42. doi: 10.1021/acsomega.4c09513 (PMC11740152; doi:10.1021/acsomega.4c09513)
Supplement: Supplementary file 1 — ao4c09513_si_001.pdf [file ao4c09513_si_001.pdf]

Semiconducting electrides derived from Sodalite: A first-principles study

Chang Liu,[†,§] Musiha Mahfuza Mukta,[‡] Byungkyun Kang,[*,¶] and Qiang Zhu[*,‡]

†*Department of Physics and Astronomy, University of Nevada, Las Vegas, NV 89154, USA*

‡*Department of Mechanical Engineering and Engineering Science, University of North Carolina at Charlotte, Charlotte, NC 28223, USA*

¶*College of Arts and Sciences, University of Delaware, Newark, Delaware 19716, USA*

§*International Center for Computational Methods & Software, College of Physics, Jilin University, Changchun, Jilin, 130012, China*

E-mail: bkang@udel.edu; qzhu8@uncc.edu

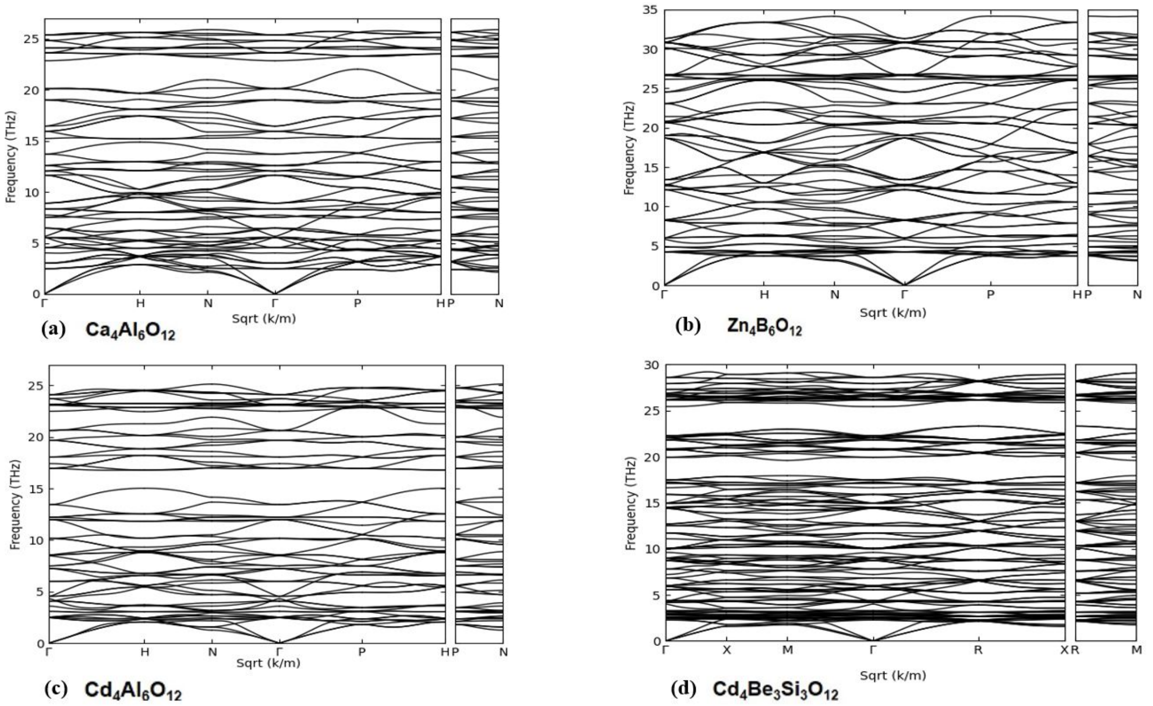

Figure S1: The calculated phonon spectra for all candidate electride phases after the removal of center O/S/Se atoms from the parental sodalites.

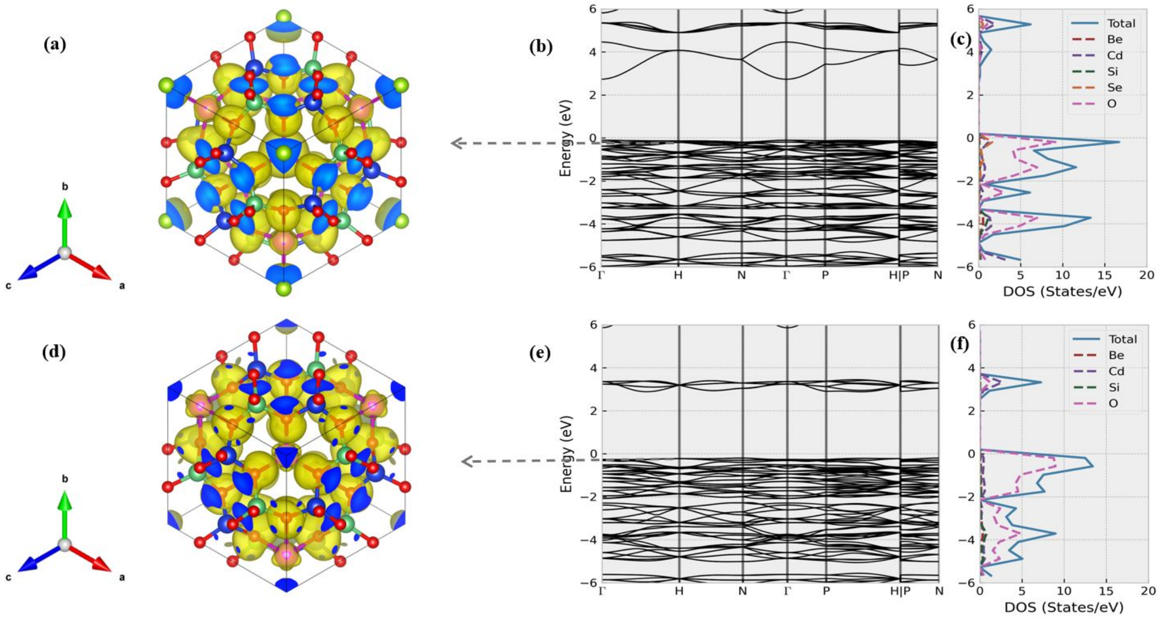

Figure S2: Electronic structures of the parent structure $Cd_4Be_3Si_3SeO_{12}$ and $Cd_4Be_3Si_3O_{12}$. (a) displays the isosurface of the decomposed charge density of $Cd_4Be_3Si_3SeO_{12}$'s highest conduction band, whereas (b) and (c) plot its band dispersion and density of states (DOS) at an extended energy range around the Fermi level. As a comparison, (d)-(f) show the isosurface of the decomposed charge density of $Cd_4Be_3Si_3O_{12}$'s highest conduction band and its corresponding band structure and DOS plots. The isosurface value is set as 0.003 e/bohr^3 in (a) and (d).

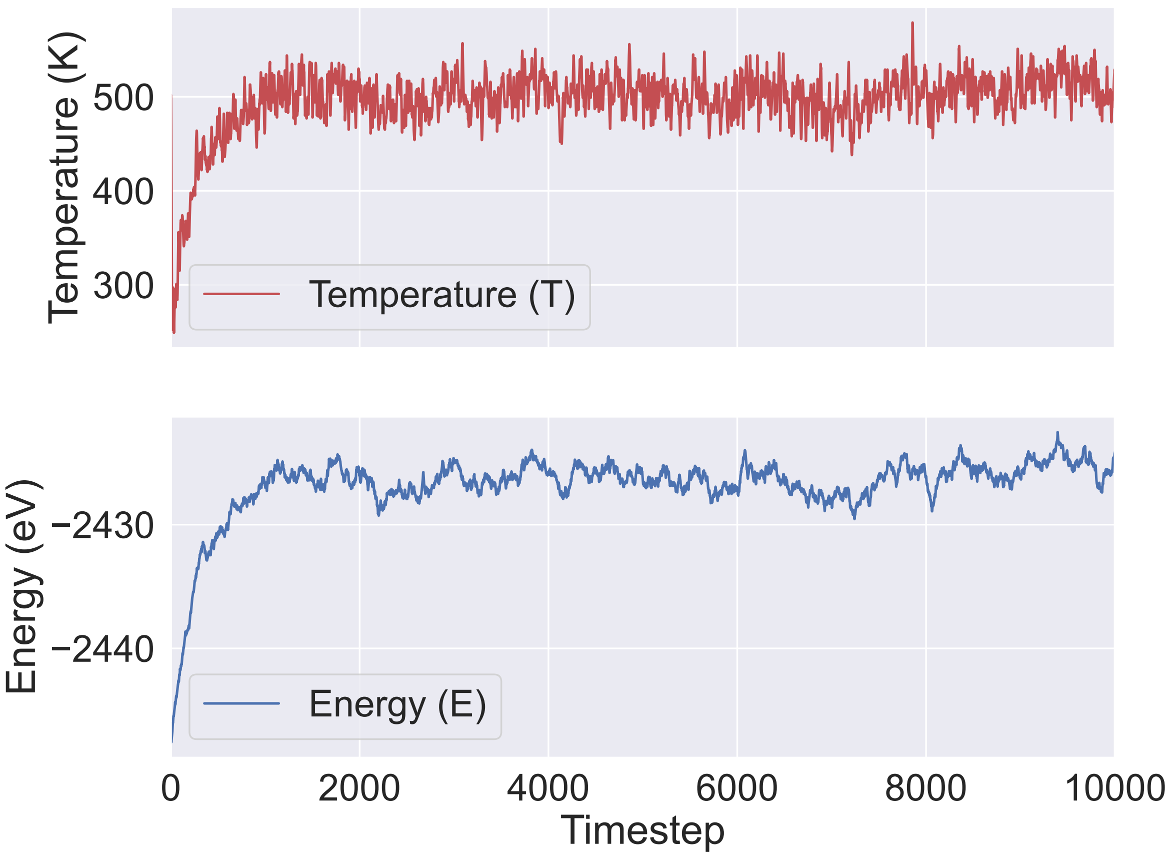

Figure S3: The on-the-fly machine learning potential based NPT MD simulation of $Ca_4Al_6O_{12}$ at 500 K. (a) shows the evolution of temperature and (b) shows the evolution of total energy.
